# Physical activity dose-response and long-term effects on dyslipidemia in Chinese adults: a CHARLS study

**DOI:** 10.1186/s12889-025-24568-1

**Published:** 2025-09-29

**Authors:** Kang Wan, Ruwen Wang, Hongmei Yan, Wei Cheng, Fuyi Ma, Yue Jin, Ru Wang

**Affiliations:** 1 School of Exercise and Health, Shanghai University of Sport, Shanghai, China; 2 Physical Education College, Henan Sport University, Zhengzhou, China; 3 Department of Endocrinology and Metabolism, Zhongshan Hospital, Fudan University, Shanghai, China; 4 Department of Endocrinology, Yangpu Hospital, School of Medicine, Tongji University, Shanghai, China

**Keywords:** Physical activity, Dyslipidemia, CHARLS, Dose-response

## Abstract

**Background:**

This longitudinal study aimed to investigate the dose-response relationship and long-term protective effects of physical activity (PA) on dyslipidemia among middle-aged and older Chinese adults, with a focus on identifying optimal PA thresholds for sustained improvements in lipid profiles.

**Methods:**

Utilizing data from the China Health and Retirement Longitudinal Study (CHARLS, 2011–2020), 3,719 participants aged ≥ 45 years were stratified into quartiles (Q1–Q4) based on weekly metabolic equivalent hours (MET-h/week). Restricted cubic spline (RCS) models and Cox proportional hazards regression adjusted for demographics, lifestyle factors, and physiological parameters were employed to assess nonlinear associations between PA dose and dyslipidemia incidence (defined by elevated TC, TG, LDL-C, or reduced HDL-C).

**Results:**

Higher PA levels demonstrated a graded reduction in dyslipidemia risk. Compared to Q1 (lowest activity), Q4 (highest activity) exhibited a 19% lower risk (fully adjusted HR = 0.81, 95% CI:0.66–0.98). RCS analysis revealed a nonlinear dose-response curve, with maximal risk reduction at 63.88–163.66 MET-h/week. Subgroup analyses confirmed consistent protective effects across genders, age groups, and BMI categories. Notably, PA exerted heterogeneous effects on lipid subcomponents: HDL-C and TG showed the strongest improvements, while LDL-C reductions plateaued at higher PA doses.

**Conclusions:**

This longitudinal study advocates metabolically-tailored PA prescriptions for dyslipidemia, with a nonlinear dose-response curve refuting “more is better” assumptions. Lipid-specific mechanisms demand differentiated exercise regimens: dose-dependent HDL-C optimization versus moderate LDL-C control. Age- and region-specific PA responsiveness underscores demographically-informed guidelines. These findings provide evidence to inform precision exercise guidelines aimed at reducing cardiovascular risk in aging populations.

## Introduction

Dyslipidemia—clinically characterized by elevated total cholesterol, low-density lipoprotein cholesterol (LDL-C), and triglycerides (TG), alongside reduced high-density lipoprotein cholesterol (HDL-C) [1, 2], represents a modifiable risk factor for global disease burden. As a primary driver of cardiovascular morbidity, it contributes significantly to atherosclerotic cardiovascular disease (ASCVD) pathogenesis [3–5]. In China alone, ASCVD accounted for 66% of cardiovascular-related deaths (2.4 million) in 2016 [6], highlighting the urgent need for effective management. Its etiology reflects a complex interplay between genetic predisposition and modifiable factors such as sedentary behavior, poor diet, obesity, and metabolic dysfunction [7–10].

Current U.S. and European guidelines prioritize statins as first-line pharmacotherapy for LDL-C reduction [11, 12]. However, statin therapy presents limitations including contraindications in pregnancy, hepatotoxicity risks, and cost barriers [13]. These constraints have prompted clinical guidelines to emphasize early lifestyle modifications—particularly increased physical activity (PA), nutritional interventions, weight control, and smoking cessation—as foundational preventive measures [14–16].

PA, defined as skeletal muscle-mediated energy expenditure exceeding resting levels [17], exerts multifaceted lipid-regulatory effects. Regular engagement reduces triglycerides by ≤ 50%, elevates HDL-C by 5–10%, and modifies LDL particle composition from atherogenic small-dense to less harmful large-buoyant subtypes [18–20]. Global recommendations endorse 3.5–7 h per week of moderate-to-vigorous PA (30–60 min daily) [21], supported by Zhang et al.‘s cross-sectional study of 17,535 Chinese adults linking low leisure-time PA to adverse lipid profiles [22].

Although the lipid benefits of PA are well-established, its dose-response dynamics resemble those of pharmacotherapy, requiring careful calibration for both efficacy and safety [23]. Notably, high-intensity PA yields lipid improvements comparable to moderate levels [24], but with heightened risks of cardiovascular strain, musculoskeletal injury, and gastrointestinal issues [25–27]. This underscores the need to define both the minimum effective and maximum safe thresholds of PA. Moreover, whether PA-induced lipid improvements are sustained long-term remains uncertain, given limited longitudinal evidence. Do different PA doses exert distinct effects or exhibit dose-gradient relationships across lipid components (TC, TG, HDL-C, LDL-C)? The literature remains inconclusive [22, 28–30].

To address these uncertainties, we conducted a 10-year longitudinal analysis (2011–2020) of Chinese adults aged ≥ 45 years using data from the China Health and Retirement Longitudinal Study (CHARLS). A restricted cubic spline (RCS) model was used to examine the dose–response relationship between physical activity (PA), measured in MET-hours/week, and dyslipidemia incidence. Cox regression and stratified models were applied to assess associations across quartiles and subgroups. The primary objective of this study is to identify the optimal range of physical activity associated with reduced dyslipidemia risk and to determine whether different lipid components exhibit distinct dose–response patterns. We hypothesize that moderate-to-vigorous PA is associated with a nonlinear, dose-dependent reduction in dyslipidemia risk, with a threshold beyond which the protective effect plateaus. These findings may help guide tailored PA recommendations for lipid management in aging populations.

## Methods

### Study design and participants

This analysis utilized data from the China Health and Retirement Longitudinal Study (CHARLS), a nationally representative cohort of adults ≥ 45 years approved by Peking University^’^s ethics board (IRB00001052-11015), with written consent from all participants. As a retrospective analysis of existing anonymized datasets, patients and the public were not involved in the design, implementation, reporting, or dissemination plans of this research. Methodological details, including sampling and eligibility criteria, were previously described [31]. Baseline data collection occurred from June 2011 to March 2012, capturing 17,708 participants from 10,257 households nationwide. These individuals participated in biennial follow-up assessments (2013, 2015, 2018, 2020) conducted via face-to-face interviews using Computer-Assisted Personal Interviewing (CAPI) systems.

**Fig. 1:**
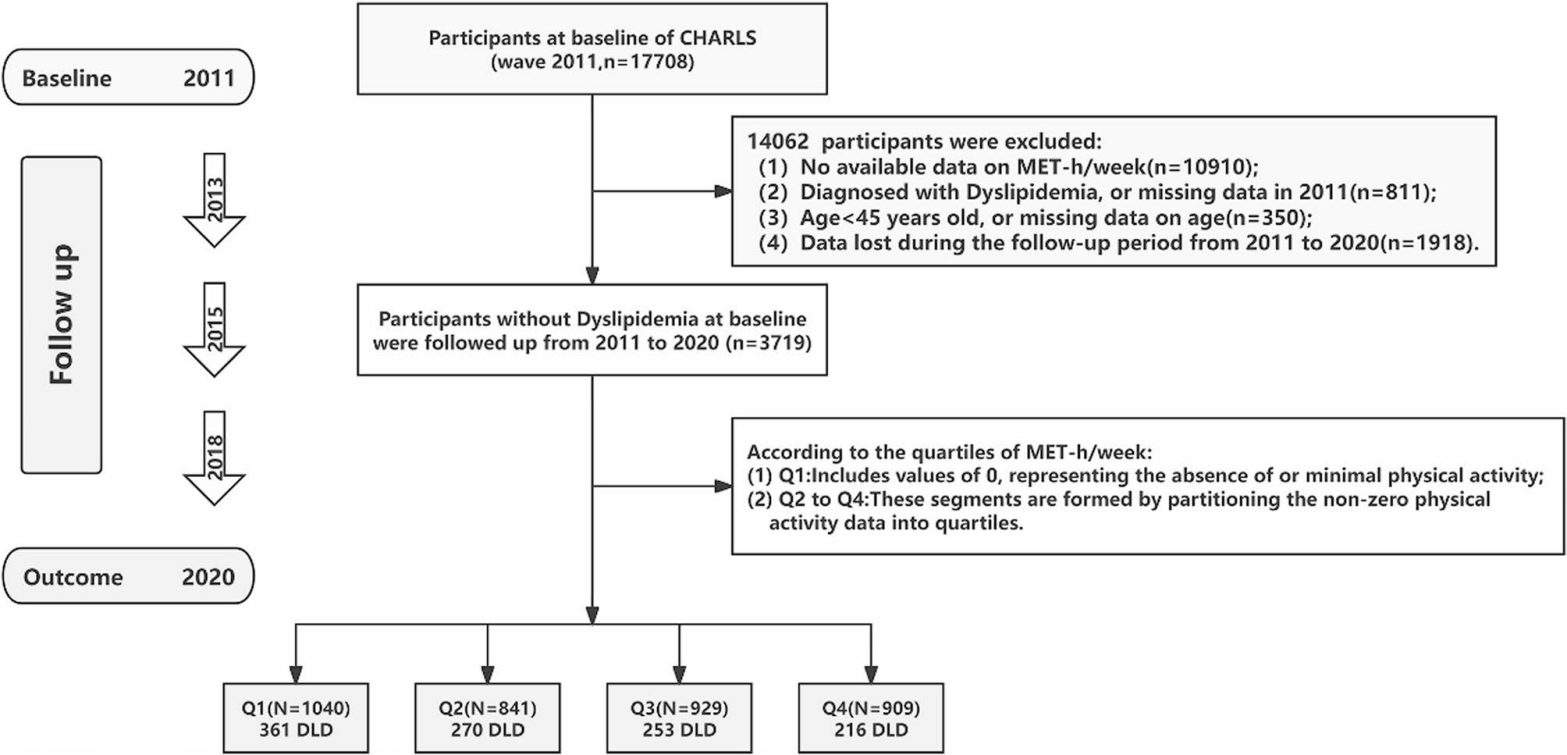
The flowchart of study participants

The analytical sample comprised 3,719 participants divided into quartiles according to their weekly metabolic equivalent of task hours (MET-h/week). As depicted in Fig. 1, exclusion criteria eliminated 13,863 initial participants based on: (1) unavailable MET-h/week measurements (*n* = 10,910) stemming from randomized physical activity question modules in 2011–2015 survey waves; (2) incomplete baseline dyslipidemia records (*n* = 811); (3) age ineligibility or missing age data (*n* = 350); and (4) loss to follow-up (*n* = 1,918).

### Variables

The CHARLS study employed standardized protocols for data collection, including blood pressure measurements (triplicate seated readings averaged after ≥ 5-min rest) and anthropometrics (height, weight, waist circumference in light clothing; BMI calculated as kg/m² with ≥ 25 defining overweight). Functional capacity was assessed using validated ADL (6-item self-care tasks, e.g., feeding, hygiene) and IADL (5-item societal skills, e.g., finances, cooking) scales, scoring 1 point per limitation (max 6 ADL/5 IADL; higher scores = greater impairment). Exercise adherence to medical advice and self-reported sleep duration were recorded. Dyslipidemia diagnosis combined healthcare-verified self-reports with clinical thresholds: elevated TG (≥ 150 mg/dL), TC (≥ 200 mg/dL), or LDL (≥ 130 mg/dL), or reduced HDL (< 40 mg/dL men/<50 mg/dL women). Chronic diseases were medically confirmed from 14 predefined conditions. These operationalized metrics enabled analysis of physical activity, functional status, and dyslipidemia risk linkages.

### Data sources and outcome determination

This longitudinal investigation examined the temporal relationship between physical activity (PA) and dyslipidemia development using data spanning from the 2011 baseline survey through either the first documented dyslipidemia event or study termination in 2020. The primary exposure metric, weekly metabolic equivalent hours (MET-h/week), was derived from an enhanced International Physical Activity Questionnaire (IPAQ) [32], that systematically evaluated three PA domains:

1. Intensity stratification (vigorous/moderate/walking);
2. Duration categorization (> 4 h, 2–4 h, 30 min–2 h, < 30 min);
3. Weekly frequency (1–7 days).

To align with CHARLS methodology while preserving cross-study comparability, we implemented three protocol adaptations:

1. Interval standardization using midpoint values (e.g., 3 h for 2-4 h category);
2. Minimum duration threshold (≥ 10 min/session per IPAQ guidelines);
3. Boundary adjustments (lower-bound truncation at 30 min, upper-limit capping at 4 h).

The MET calculation framework operationalized intensity-specific multipliers:

1. 3.3 METs for walking × daily minutes × weekly days.
2. 4.0 METs for moderate activity × daily minutes × weekly days.
3. 8.0 METs for vigorous activity × daily minutes × weekly days.

Summation of these components yielded the comprehensive MET-h/week metric [33], calculated as: Total PA = Σ(Walking + Moderate + Vigorous MET-h/week).

### Sensitivity analysis and bias

To assess the potential impact f overreporting in self-reported physical activity (PA) data, we conducted a sensitivity analysis by excluding participants in the top 5% of weekly MET-h distributions (> 377.3 MET-h/week) [34]. After removing these extreme values, the analytical sample decreased from 3,719 to 3,536 participants. In the full dataset, the dyslipidemia incidence was 29.58% (1,100 out of 3,719). After exclusion of the top 5%, the dyslipidemia rate remained virtually unchanged at 29.95% (1,059 out of 3,536). Similarly, the number of non-dyslipidemia cases only slightly changed from 2,619 to 2,477.

These findings suggest that the highest-reported physical activity values—potentially subject to recall bias or overestimation—did not materially bias the observed associations. This reinforces the robustness of the relationship between PA and dyslipidemia, despite inherent limitations of self-reported IPAQ-derived MET-h measures.

### Statistical methods

All analyses were performed in R statistical environment (version 4.3.0) with statistical significance defined as two-tailed *p* < 0.05. Continuous variables were characterized using appropriate measures of central tendency and dispersion: normally distributed parameters reported as mean ± standard deviation, and non-normally distributed variables expressed as median (interquartile range). Distribution normality was verified through Shapiro-Wilk testing, with parametric comparisons conducted via ANOVA and non-parametric analyses using Kruskal-Wallis H-test. Categorical data were presented as counts with proportions (%) and analyzed using χ² tests. Missing data were handled through multiple imputation techniques to maintain analytical integrity while reducing potential bias.

Kaplan-Meier methodology generated cumulative incidence curves for dyslipidemia development, stratified by baseline physical activity quartiles. Three sequential Cox proportional hazards models were developed to examine MET-h/week-dyslipidemia associations:

- Crude Model: Unadjusted baseline analysis.
- Demographic-Adjusted Model: Controlled for age, gender, marital status, residence, smoking/alcohol consumption, educational attainment, chronic disease status, and baseline exercise patterns.
- Full Model: Further adjusted for physiological parameters (blood pressure, waist circumference, BMI), sleep duration, and functional capacity scores (ADL/IADL).

Multicollinearity was systematically evaluated through variance inflation factors (VIF < 10 for all covariates). Stratified analyses examined effect modification across key subgroups: age dichotomization (< 60 vs. ≥ 60 years), gender, smoking/drinking status, marital status, urban/rural residence, and BMI categories (< 25 vs. ≥ 25 kg/m²). These stratification procedures enabled assessment of association robustness across population subsets.

Restricted cubic spline (RCS) regression with four knots (5th, 35th, 65th, 95th percentiles) elucidated potential nonlinear dose-response relationships. To investigate the dose–response relationship between MET-h/week and the incidence of dyslipidemia, RCS based on Cox regression models was employed, adjusting covariates in model 3, and the MET-h/week value at HR = 1 was treated as the reference. All regression models satisfied proportional hazards assumptions and demonstrated adequate goodness-of-fit through diagnostic testing.

## Results

### Baseline characteristics of participants

The analytical cohort comprised 3,719 subjects, stratified into quartiles (Q1-Q4) based on weekly metabolic equivalent hours (MET-h/week). As shown in Table 1, significant interquartile differences were observed across demographic, physiological, and health status parameters (all *P* < 0.05 unless otherwise specified). Stratification by activity levels revealed substantial variations in baseline characteristics, with an inverse relationship between MET-h/week quartiles and participant age (*P* < 0.001). Participants in the most active quartile (Q4) demonstrated the youngest mean age.

**Table 1:** Baseline characteristics of participants stratified by quartiles of METs

Characteristics	Overall (*n* = 3719)	Quartiles of mets				*P *value
		Q1 (*n* = 1040)	Q2 (*n* = 841)	Q3 (*n* = 929)	Q4 (*n* = 909)	
Met, h/week	104.30 (28.88, 224.00)	7.70 (0.00,28.88)	69.30 (57.38,84.00)	153.30 (119.70,185.00)	328.30 (280.88,368.40)	< 0.001
Dyslipidemia, *n* (%)	1100 (29.58)	361 (34.71)	270 (32.10)	253 (27.23)	216 (23.76)	< 0.001
Age, years	58.21 ± 8.62	60.43 ± 9.60	58.53 ± 8.73	57.22 ± 7.82	56.37 ± 7.47	< 0.001
SBP, mmHg	127.98 ± 20.83	129.98 ± 21.67	128.19 ± 20.72	127.17 ± 20.61	126.33 ± 20.01	< 0.001
DBP, mmHg	74.91 ± 11.95	76.02 ± 12.03	74.85 ± 11.60	74.34 ± 12.02	74.28 ± 12.05	0.003
Weight, kg	58.91 ± 11.50	60.06 ± 12.33	59.58 ± 11.16	58.42 ± 11.34	57.48 ± 10.78	< 0.001
Waist, cm	84.28 ± 12.21	86.60 ± 12.15	84.53 ± 13.03	83.56 ± 12.08	82.14 ± 11.12	< 0.001
Bmi, kg/m^2^	23.54 ± 3.84	24.04 ± 4.10	23.83 ± 3.76	23.41 ± 3.78	22.82 ± 3.5	< 0.001
Sleep, h	6.43 ± 1.84	6.36 ± 1.89	6.43 ± 1.84	6.42 ± 1.88	6.51 ± 1.75	0.348
Female, *n* (%)	2037 (54.77)	623 (59.90)	485 (57.67)	523 (56.30)	406 (44.66)	< 0.001
Drinking, *n* (%)	1193 (32.08)	268 (25.77)	243 (28.89)	300 (32.29)	382 (42.02)	< 0.001
Smoking, *n* (%)	1122 (29.18)	269 (25.19)	216 (24.63)	279 (29.00)	358 (38.17)	< 0.001
Married, *n* (%)	3354 (90.19)	897 (86.25)	751 (89.30)	855 (92.03)	851 (93.62)	< 0.001
Rural residence, *n* (%)	2437 (65.53)	573 (55.10)	482 (57.31)	652 (70.18)	730 (80.31)	< 0.001
Education, *n* (%)						< 0.001
Illiterate	1726 (46.41)	458 (44.04)	360 (42.81)	456 (49.09)	452 (49.72)	
Primary school	803 (21.59)	228 (21.92)	168 (19.98)	202 (21.74)	205 (22.55)	
Middle school	786 (21.13)	229 (22.02)	185 (22.00)	187 (20.13)	185 (20.35)	
High school and above	404 (10.86)	125 (12.02)	128 (15.22)	84 (9.04)	67 (7.37)	
Exercise, *n* (%)	3367 (90.54)	688 (66.15)	841 (100.00)	929 (100.00)	909 (100.00)	< 0.001
Chronic, *n* (%)	2461 (64.01)	691(64.70)	560(63.85)	632(65.70)	578(61.62)	0.188
6 ADL diff, *n* (%)						< 0.001
0	3164 (85.08)	833 (80.10)	731 (86.92)	802 (86.33)	798 (87.79)	
1	289 (7.77)	85 (8.17)	58 (6.90)	81 (8.72)	65 (7.15)	
2	122 (3.28)	40 (3.85)	28 (3.33)	26 (2.80)	28 (3.08)	
3	52 (1.40)	23 (2.21)	14 (1.66)	7 (0.75)	8 (0.88)	
4	44 (1.18)	29 (2.79)	5 (0.59)	6 (0.65)	4 (0.44)	
5	33 (0.89)	19 (1.83)	5 (0.59)	6 (0.65)	3 (0.33)	
6	15 (0.40)	11 (1.06)	0 (0.00)	1 (0.11)	3 (0.33)	
5 IADL diff, *n* (%)						< 0.001
0	3006 (80.83)	772 (74.23)	708 (84.19)	765 (82.35)	761 (83.72)	
1	327 (8.79)	88 (8.46)	65 (7.73)	97 (10.44)	77 (8.47)	
2	183 (4.92)	64 (6.15)	36 (4.28)	41 (4.41)	42 (4.62)	
3	104 (2.80)	46 (4.42)	22 (2.62)	17 (1.83)	19 (2.09)	
4	60 (1.61)	39 (3.75)	6 (0.71)	5 (0.54)	10 (1.10)	
5	39 (1.05)	31 (2.98)	4 (0.48)	4 (0.43)	0 (0.00)	

*BMI* Body mass index; *Sleep* Self-reported average daily hours of sleep over the past month; *SBP* Systolic blood pressure; *DBP* Diastolic bloodpressure; *MET* Metabolic equivalent of task; Recommended Exercise: ever received medical exercise advice? Functional limitations were quantified using:ADL (Activities of Daily Living) score (0-6 scale assessing six basic daily tasks) and IADL (Instrumental Activities of Daily Living) score (0-5 scaleevaluating complex life-management activities)

Physiological profiling revealed progressive improvements across activity quartiles in various cardiometabolic indicators: systolic blood pressure (129.98 ± 21.67 vs. 128.19 ± 20.72 mmHg, Q1 vs. Q4), diastolic blood pressure (76.02 ± 12.03 vs. 74.18 ± 12.05 mmHg), body weight (60.06 ± 12.33 vs. 57.48 ± 10.78 kg), waist circumference (86.60 ± 12.15 vs. 82.14 ± 11.12 cm), and BMI (24.04 ± 4.10 vs. 22.82 ± 3.50 kg/m²), all showing significant decreases with increasing activity levels (*P* < 0.001–0.003). Sleep duration remained comparable across quartiles (*P* = 0.348).

Population stratification indicated distinct demographic patterns associated with activity intensity. Higher MET-h/week quartiles contained proportionally more males, alcohol consumers, smokers, married individuals, and rural residents (all *P* < 0.001). Conversely, educational attainment exhibited an inverse relationship with activity levels, with lower proportions of highly educated participants in the upper quartiles.

Health outcome analysis revealed two divergent patterns: chronic disease prevalence showed no interquartile variation (*P* = 0.188), while dyslipidemia rates demonstrated a significant dose-dependent reduction across activity quartiles (34.71% vs. 23.76%, Q1 vs. Q4; *P* < 0.001). Functional capacity assessments through ADL/IADL scales indicated superior performance in higher activity groups, with Q4 participants reporting 87.79% ADL independence versus 80.10% in Q1 (*P* < 0.001). Clinician-initiated exercise recommendations showed quartile-dependent adoption rates, increasing from 66.15% in Q1 to 100% in the upper quartiles (*P* < 0.001).

### Cumulative incidence and dose–response relationship between physical activity and dyslipidemia

**Fig. 2:**
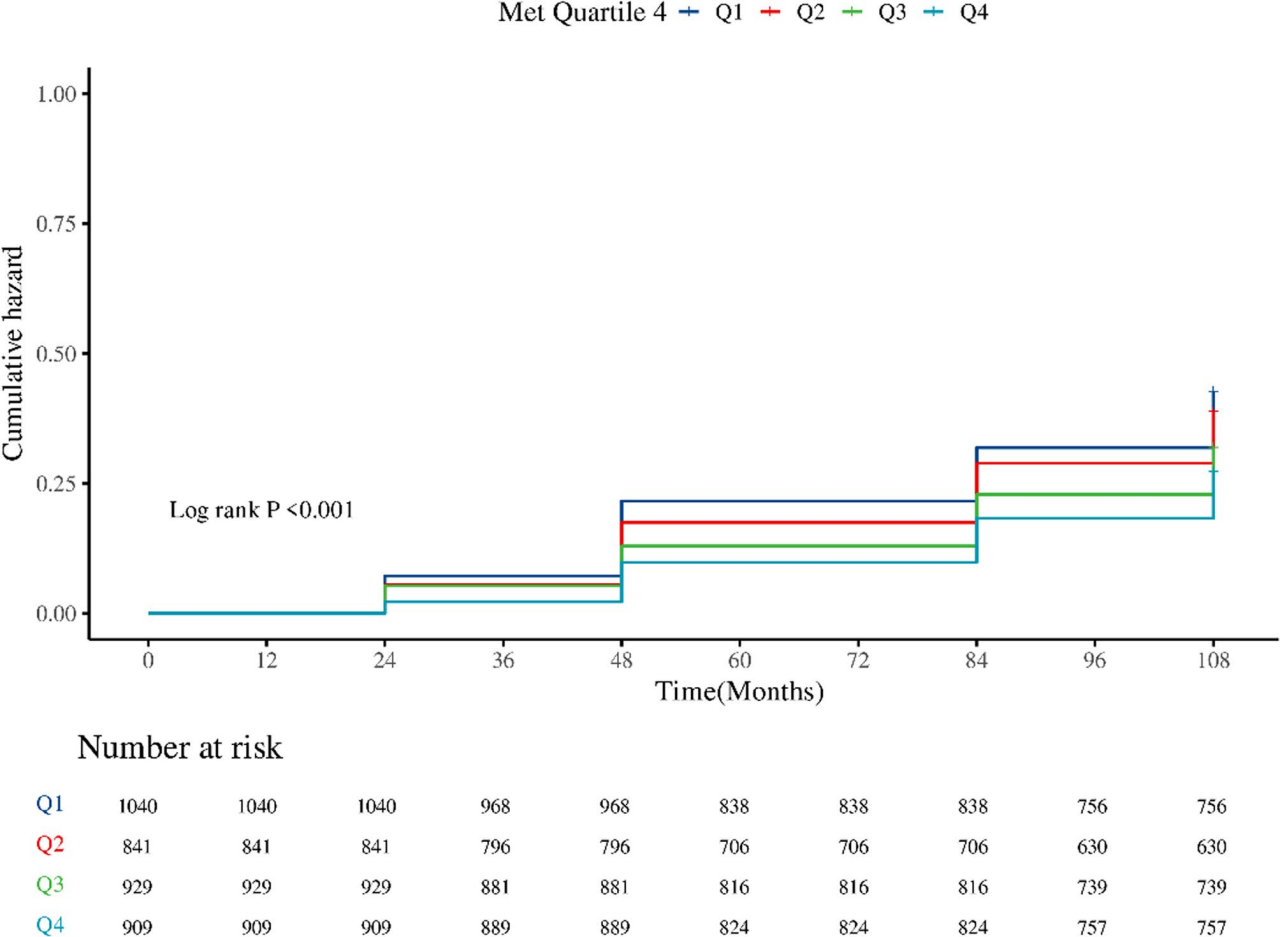
Kaplan–Meier curves for the cumulative incidence of dyslipidemia by baseline physical activity quartiles

Kaplan–Meier survival curve analysis showed a graded inverse association between baseline physical activity levels (MET-h/week) and cumulative incidence of dyslipidemia. Participants in the highest activity quartile (Q4) consistently exhibited the lowest cumulative incidence across the follow-up period, while those in the lowest quartile (Q1) had the highest. Divergence between curves appeared early during follow-up (Fig. 2) and widened progressively over time. At 108 months, the difference between Q1 and Q4 reached its maximum, with intermediate quartiles (Q2–Q3) displaying proportional, stepwise reductions in risk.

**Fig. 3:**
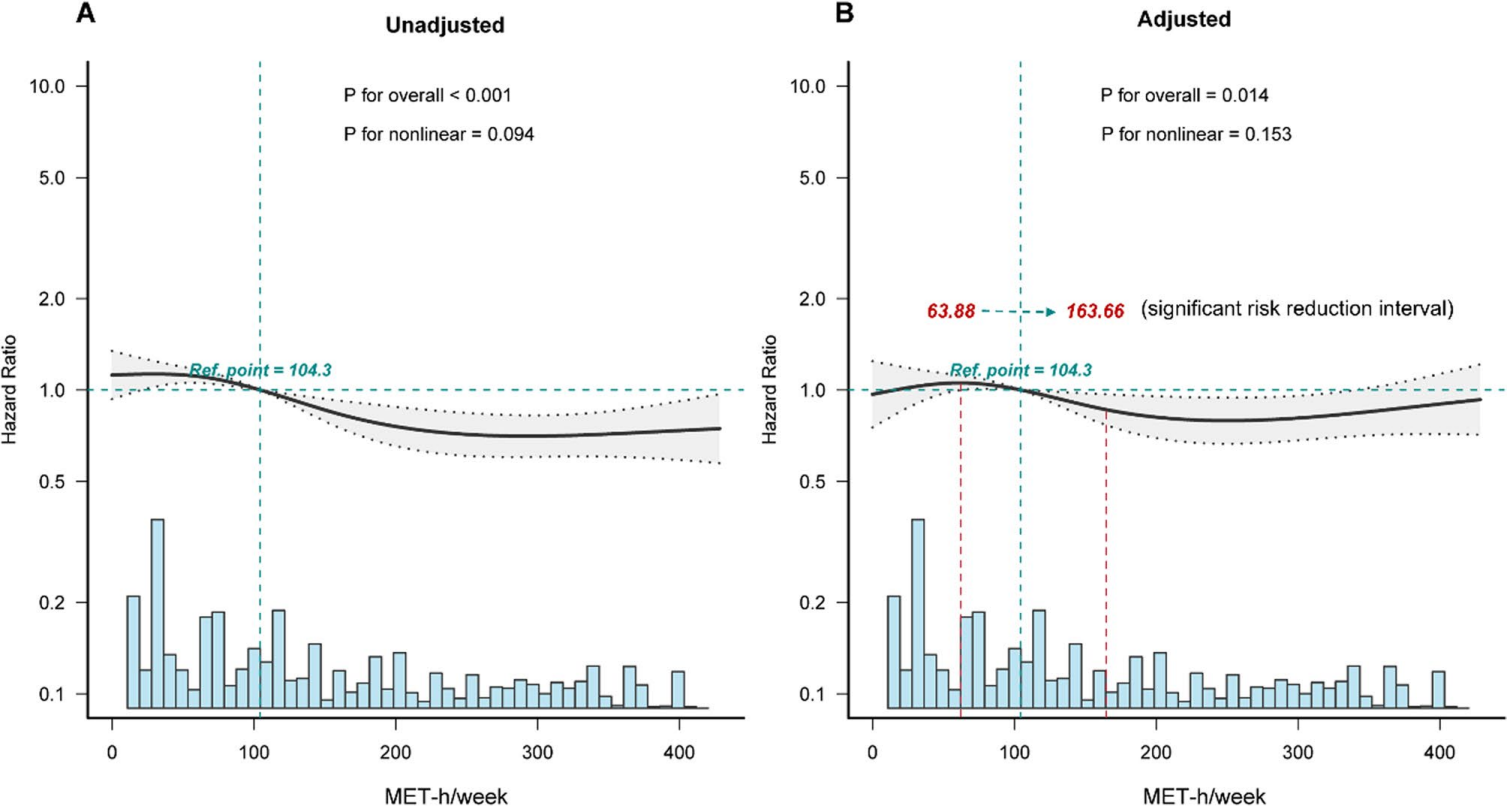
Unadjusted (Fig. 3A): significant negative association (*P* < 0.001), strongest reduction at 63.88–163.66 MET-h/week, plateau with minor resurgence (*P* = 0.094 for nonlinearity). Adjusted (Fig. 3A): retained significance(*P* = 0.014) but nonsignificant nonlinearity (*P* = 0.153)

Using restricted cubic spline (RCS) regression, we examined the nonlinear dose-response relationship between MET-h/week and dyslipidemia risk. Nodes were placed at the 5th (0 MET-h/week), 35th (63.88 MET-h/week), 65th (163.66 MET-h/week), and 95th (377.5 MET-h/week) percentiles of the MET-h/week distribution, with 104.3 MET-h/week (HR = 1) set as the reference value. The analysis revealed a significant overall negative association between total MET-h/week and dyslipidemia risk (*P*_for overall_ < 0.001). The strongest risk reductions were observed in the range of 63.88–163.66 MET-h/week, with the risk plateauing at higher activity levels and showing a minor resurgence (As shown in Fig. 3A). After adjusting for all confounders, the negative association remained significant (*P*_for overall_ = 0.014), although nonlinearity was not statistically significant (*P*_for non−linearity_ = 0.153; As shown in Fig. 3B).

### Cox proportional hazards regression

In the Cox proportional hazards models, higher physical activity levels (MET-h/week) were associated with progressively lower dyslipidemia risk across quartiles. In the unadjusted model (Model 1), hazard ratios (HRs) for Q2, Q3, and Q4 compared with Q1 were 0.90 (95% CI: 0.77–1.05, *P* = 0.186), 0.73 (95% CI: 0.63–0.86, *P* < 0.001), and 0.62 (95% CI: 0.52–0.73, *P* < 0.001), respectively, with significant risk reduction from Q3 onward. After adjusting for demographic and behavioral covariates (Model 2), HRs were 0.80 (0.67–0.96, *P* = 0.002) for Q3 and 0.74 (0.61–0.90, *P* = 0.001) for Q4.

In the fully adjusted model (Model 3), which additionally accounted for physiological parameters and functional status scores, Q4 maintained a significant association (HR = 0.81, 95% CI: 0.66–0.98, *P* = 0.035). Q3 showed a non-significant trend (HR = 0.85, 0.71–1.02, *P* = 0.088), and Q2 remained neutral (HR = 0.97, 0.81–1.16, *P* = 0.714). The dose-response pattern across quartiles persisted. Per standard deviation increase in MET-h/week, HRs were 0.83 (0.77–0.88, *P* < 0.001) in Model 1, 0.89 (0.83–0.95, *P* = 0.001) in Model 2, and 0.92 (0.85–0.99, *P* = 0.019) in Model 3, indicating a consistent inverse association across all models (Table 2).

**Table 2:** Multivariate-adjusted hazard ratios of MET-h/week for dyslipidemia

Variables	Total *N* No. of events (Incident rate)		Model 1		Model 2		Model 3	
			HR (95%CI)	*P*	HR (95%CI)	*P*	HR (95%CI)	*P*
Per SD increase	3719	1100(29.6)	0.83(0.77 ~ 0.88)	< 0.001	0.89(0.83 ~ 0.95)	0.001	0.92 (0.85 ~ 0.99)	0.019
Q1	1040	361(34.7)	1.00 (Reference)		1.00 (Reference)		1.00 (Reference)	
Q2	841	270(32.1)	0.90 (0.77 ~ 1.05)	0.186	0.92 (0.77 ~ 1.10)	0.37	0.97 (0.81 ~ 1.16)	0.714
Q3	929	253(27.2)	0.73 (0.63 ~ 0.86)	< 0.001	0.80 (0.67 ~ 0.96)	0.002	0.85 (0.71 ~ 1.02)	0.088
Q4	909	216(23.8)	0.62(0.52 ~ 0.73)	< 0.001	0.74 (0.61 ~ 0.90)	0.001	0.81 (0.66 ~ 0.98)	0.035

*HR* Hazard ratio; *CI* Confidence interval

Model1: unadjusted

Model2: Adjust: gender, age, marital status, rural residence, drinking, smoking, education, chronic, recommended exercise

Model3: Adjust: gender, age, marital status, rural residence, drinking, smoking, education, chronic, recommended exercise, SBP, DBP, weight, waist, BMI, sleep, ADL difficulty Score, IADL difficulty score

### Subgroup analyses

Using the threshold of 104.3 MET-h/week identified through restricted cubic spline (RCS) regression (as shown in Table 3), physical activity levels were dichotomized into Light Physical Activity (LPA: <104.3 MET-h/week) and Moderate-to-Vigorous Physical Activity (MVPA: ≥104.3 MET-h/week). The analysis showed that sustained MVPA significantly reduced dyslipidemia risk, with a hazard ratio (HR) of 0.74 (95% CI: 0.66–0.83; *P* < 0.001).

**Table 3:** Subgroup analysis of the association between MET-h/week and dyslipidemia

Variables	*n* (%)	LPA	MVPA	HR (95%CI)	*P*	*P* for interaction
All patients	3719 (100.00)	616/1845	484/1874	0.73 (0.65 ~ 0.83)	< 0.001	
Gender						0.219
Female	2037 (54.77)	381/1085	245/952	0.69 (0.59 ~ 0.81)	< 0.001	
Male	1682 (45.23)	235/760	239/922	0.80 (0.67 ~ 0.96)	0.018	
Age						0.751
< 60	2209 (59.40)	328/996	306/1213	0.73 (0.62 ~ 0.85)	< 0.001	
≥ 60	1510 (40.60)	288/849	178/661	0.76 (0.63 ~ 0.91)	0.003	
Drinking						0.848
No	2526 (67.92)	468/1344	323/1182	0.74 (0.64 ~ 0.85)	< 0.001	
Yes	1193 (32.08)	148/501	161/692	0.76 (0.61 ~ 0.95)	0.016	
Smoking						0.681
No	2615 (70.31)	477/1380	330/1235	0.73 (0.64 ~ 0.84)	< 0.001	
Yes	1104 (29.69)	139/465	154/639	0.77 (0.61 ~ 0.97)	0.028	
Residence						0.648
Rural	2437 (65.53)	317/1037	336/1400	0.75 (0.65 ~ 0.88)	< 0.001	
Urban	1282 (34.47)	299/808	148/474	0.81 (0.66 ~ 0.98)	0.033	
Bmi						0.170
< 25	2563 (68.92)	344/1200	293/1363	0.71 (0.61 ~ 0.83)	< 0.001	
≥ 25	1156 (31.08)	272/645	191/511	0.85 (0.71 ~ 1.02)	0.084	

*HR* Hazard ratio; *CI* Confidence interval

Stratified analyses revealed consistent protective effects of MVPA across most subgroups. Significant risk reductions were observed in both males (HR = 0.80, *P* = 0.015) and females (HR = 0.71, *P* < 0.001), participants aged < 60 years (HR = 0.74, *P* < 0.001) and ≥ 60 years (HR = 0.76, *P* = 0.003), as well as across drinking status, smoking status, and residential areas (urban/rural). No significant interaction effects were detected between MVPA and these stratification variables (*P* interaction > 0.05 for all), indicating robust protective associations regardless of demographic or behavioral characteristics.

The risk reduction associated with MVPA did not reach statistical significance in unmarried participants (HR = 0.85, *P* = 0.403). This finding may reflect limited statistical power due to smaller subgroup size (*n* = 368 unmarried vs. *n* = 3,477 married), or potential confounding by differential social support patterns and health behaviors between marital status groups.

### Dose–response analysis of lipid subcomponents

Building on previous findings from 2011 to 2020 (*N* = 3,791), which demonstrated that physical activity exceeding guideline-recommended thresholds significantly reduces dyslipidemia risk—albeit with plateauing benefits at higher doses—this study investigates the differential effects of exercise intensity on lipid subcomponents (TC, TG, HDL-C, LDL-C). By narrowing the observational window to 2011–2015 (capturing two timepoints with complete lipid profiles in the CHARLS database), we analyzed 2,710 eligible participants after excluding 1,081 cases with incomplete or substandard data. Participants were stratified into quartiles based on weekly physical activity levels (MET-h/week), from Q1 (least active) to Q4 (most active). Biomarker-specific dose-response patterns were observed:

For total cholesterol (TC), mean concentrations decreased non-linearly from 189.29 ± 40.45 mg/dL in Q1 to 183.26 ± 34.60 mg/dL in Q2, 184.02 ± 34.43 mg/dL in Q3, and 183.67 ± 40.69 mg/dL in Q4 (*P* = 0.007). Post hoc comparisons indicated significant differences between Q1 and all other quartiles (*P* < 0.05), whereas no differences were observed among Q2–Q4 (*P* > 0.05).

Triglyceride (TG) levels declined from 146.50 ± 87.08 mg/dL in Q1 to 134.38 ± 94.87 mg/dL in Q4, with significance reached only between Q1 and Q4 (*P* = 0.0128). The prevalence of hypertriglyceridemia decreased linearly from 34.92% in Q1 to 25.04% in Q4 (*P* < 0.001).

High-density lipoprotein cholesterol (HDL-C) increased from 50.13 ± 10.66 mg/dL in Q1 to 54.47 ± 13.78 mg/dL in Q4 (*P* < 0.001), with all pairwise comparisons between adjacent quartiles being significant except Q1–Q2 (*P* > 0.05).

**Fig. 4:**
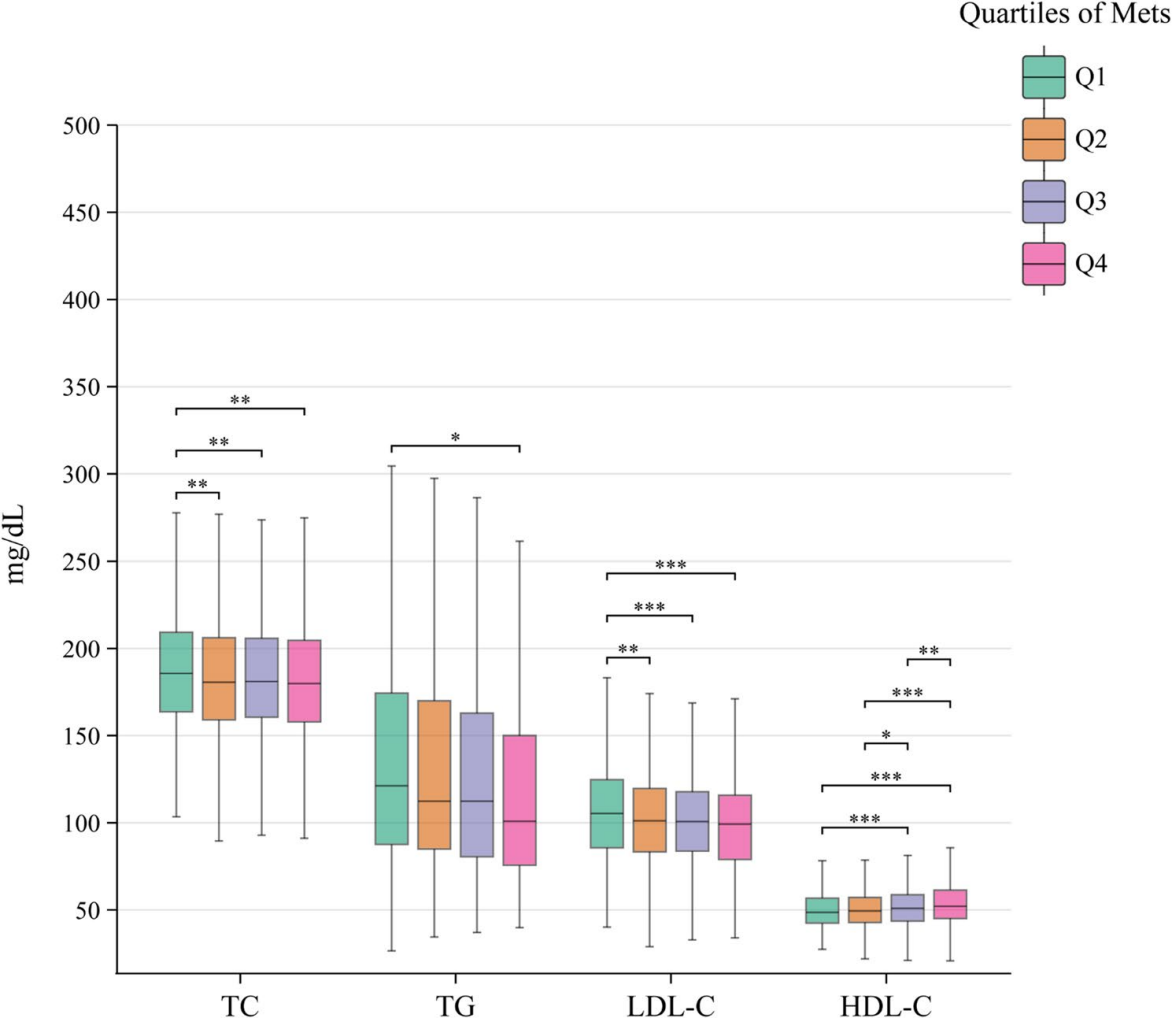
Dose-response relationships between physical activity quartiles and lipid subcomponents

Low-density lipoprotein cholesterol (LDL-C) exhibited a significant inverse association with physical activity, with mean levels of 107.40 ± 30.88 mg/dL in Q1, 102.32 ± 27.30 mg/dL in Q2, 101.61 ± 26.61 mg/dL in Q3, and 100.08 ± 29.98 mg/dL in Q4. Significant reductions were observed between Q1 and each higher quartile (all *P* < 0.05), whereas differences among Q2–Q4 were non-significant (*P* > 0.05). The above results are shown in Table 4; Fig. 4.

**Table 4:** Dose-response comparison of Q1- Q4 on lipid metabolic markers

Variables	Total (*n* = 2710)	Q1 (*n* = 733)	Q2 (*n* = 625)	Q3 (*n* = 685)	Q4 (*n* = 667)	*P*
TC, (mg/dL)	185.18 ± 37.82	189.29 ± 40.45	183.26 ± 34.60	184.02 ± 34.43	183.67 ± 40.69	0.007
TG, (mg/dL)	139.73 ± 88.64	146.50 ± 87.08	138.60 ± 81.32	138.72 ± 90.14	134.38 ± 94.87	0.075
HDL-C, (mg/dL)	51.93 ± 12.18	50.13 ± 10.66	50.75 ± 11.20	52.45 ± 12.48	54.47 ± 13.78	< 0.001
LDL-C, (mg/dL)	102.96 ± 28.93	107.40 ± 30.88	102.32 ± 27.30	101.61 ± 26.61	100.08 ± 29.98	< 0.001

**Fig. 5:**
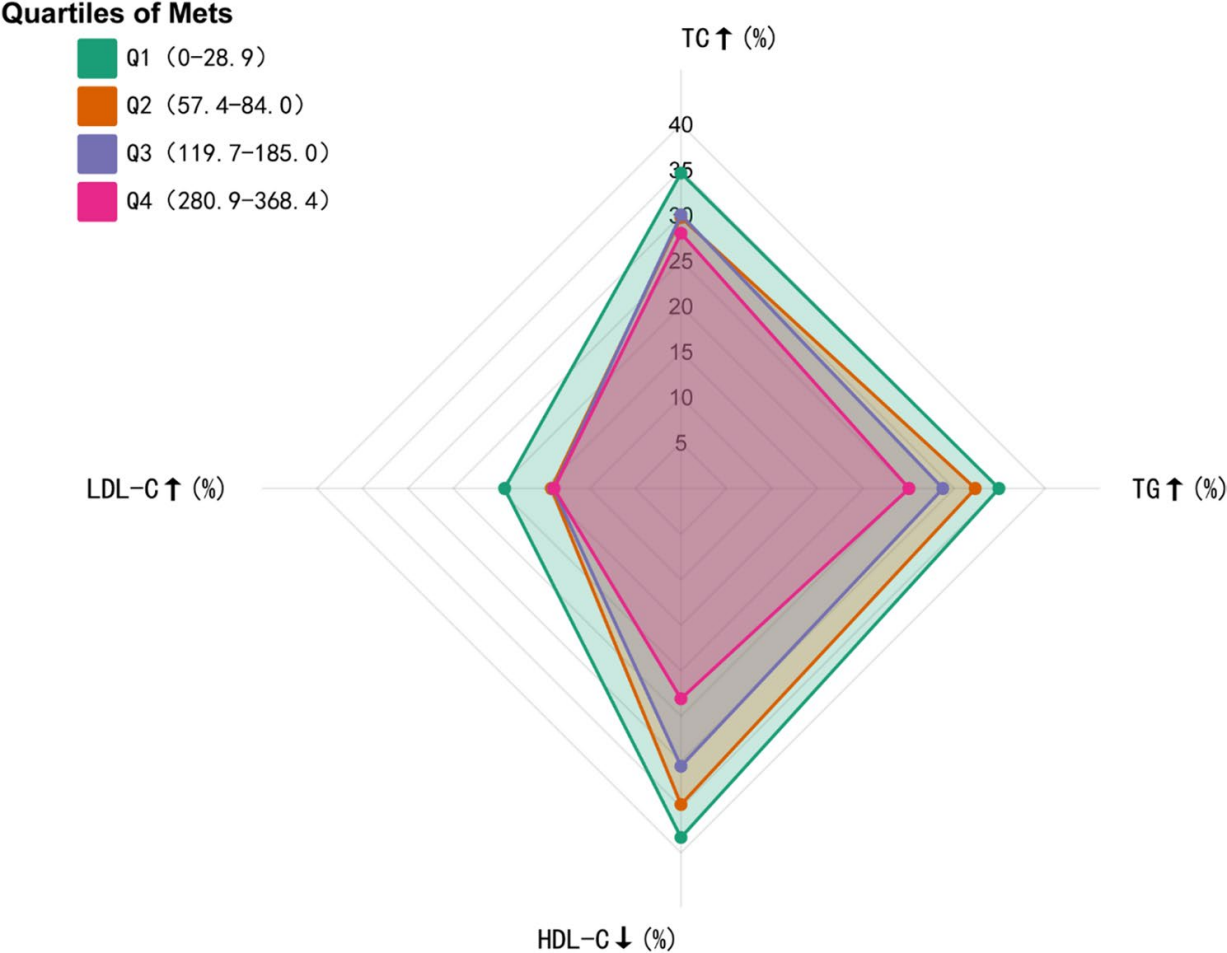
Quartile-specific distribution of dyslipidemia across metabolic equivalent of task (MET)-hours per week

In the quartile-based analysis(Q1-Q4 by MET-h/week), significant heterogeneity was observed in the prevalence of dyslipidemia(TC ≥ 200 mg/dL, TG ≥ 150 mg/dL, LDL-C ≥ 130 mg/dL, HDL-C < 39/50 mg/dL(defined as < 40 mg/dL in men and < 50 mg/dL in women); all *P* < 0.05) [35]. The proportion of HDL-C deficiency showed a clear stepwise decline from 38.34% in Q1 to 23.09% in Q4 (*P* < 0.001), accompanied by a parallel reduction in TG abnormalities (34.92–25.04%). TC abnormalities followed a generally downward trend but with slight fluctuation, while LDL-C prevalence was highest in Q1 and dropped to a stable, lower level from Q2 onwards (as shown in Table 5; Fig. 5).

**Table 5:** Gradient trends in lipid abnormality prevalence across quartile groups

Variables (mg/dL)	Total (*n* = 2710)	Q1 (*n* = 733)	Q2 (*n* = 625)	Q3 (*n* = 685)	Q4 (*n* = 667)	*P*
TC ≥ 200, *n* (%)	833 (30.74)	254 (34.65)	186 (29.76)	206 (30.07)	187 (28.04)	0.046
TG ≥ 150, *n* (%)	822 (30.33)	256 (34.92)	202 (32.32)	197 (28.76)	167 (25.04)	< 0.001
LDLC ≥ 130, *n* (%)	420 (15.50)	142 (19.37)	89 (14.24)	96 (14.01)	93 (13.94)	0.009
HDL-C<39/50, *n* (%)	861 (31.77)	281 (38.34)	217 (34.72)	209 (30.51)	154 (23.09)	< 0.001

**Fig. 6:**
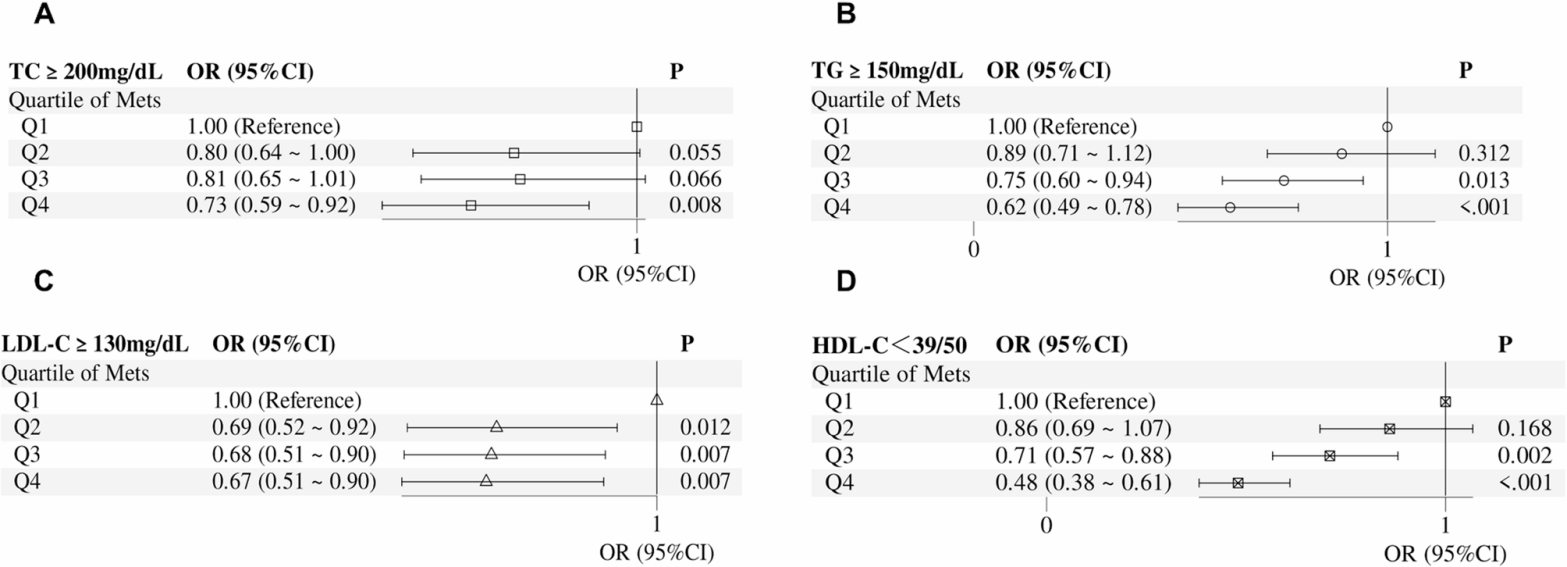
Association between quartiles of Mets and risk of dyslipidemia components

Figure 6 presents the association between quartiles of MET-h/week (with Q1 as the reference group) and the risk of different types of dyslipidemia, as indicated by odds ratios (OR) with 95% confidence intervals (CI) and P values. For total cholesterol (TC ≥ 200 mg/dL), the OR values gradually decrease with increasing MET quartiles: Q2 shows an OR of 0.80 (95% CI: 0.64–1.00) with *P* = 0.055, Q3 has an OR of 0.81 (95% CI: 0.65–1.01) with *P* = 0.066, and Q4 exhibits a statistically significant OR of 0.73 (95% CI: 0.59–0.92) with *P* = 0.008. Regarding low-density lipoprotein cholesterol (LDL-C ≥ 130 mg/dL), significant risk reduction is observed starting from Q2: Q2 has an OR of 0.69 (95% CI: 0.52–0.92) with *P* = 0.012, Q3 shows an OR of 0.68 (95% CI: 0.51–0.90) with *P* = 0.007, and Q4 has an OR of 0.67 (95% CI: 0.51–0.90) with *P* = 0.007, with relatively stable effects across Q2 to Q4. For triglycerides (TG ≥ 150 mg/dL), Q3 and Q4 show significant risk reduction: Q3 has an OR of 0.75 (95% CI: 0.60–0.94) with *P* = 0.013, and Q4 exhibits a stronger effect with an OR of 0.62 (95% CI: 0.49–0.78) and *P* < 0.001. For high-density lipoprotein cholesterol (HDL-C < 39/50 mg/dL), Q3 and Q4 also show significant protective effects: Q3 has an OR of 0.71 (95% CI: 0.57–0.88) with *P* = 0.002, and Q4 has the most pronounced effect with an OR of 0.48 (95% CI: 0.38–0.61) and *P* < 0.001. Overall, the results indicate differential effects of physical activity on various lipid parameters, with stronger dose-dependent protective effects on TG and HDL-C, stable effects on LDL-C from moderate activity levels, and significant effects on TC only at the highest activity level.

## Discussion

This 10-year CHARLS cohort analysis confirms a robust inverse association between baseline physical activity (PA) and dyslipidemia risk, with benefits increasing in a dose-dependent manner before plateauing—consistent with domestic and international findings. Our results align with Qinpei Zou et al. and prior evidence linking occupational activity to higher HDL-C. Beyond lipid regulation, high-activity participants also had better blood pressure, body weight, waist circumference, and BMI, key markers for cardiometabolic health [28].

Multiple biological pathways may explain the graded benefits of PA observed here. Skeletal muscle–derived IL-6 can suppress hypothalamic AgRP neuronal activity, reducing hunger signals and promoting fat oxidation, which may contribute to the lower BMI and waist circumference in highly active participants [36–38]. The strongest risk reduction occurred at 63.88–163.66 MET-h/week, suggesting a metabolic “saturation” threshold. This suggests that high levels of physical activity may reach a physiological threshold, such as saturation of lipid oxidation or transport pathways, leading to a plateau in effects. Additionally, excessive exercise may induce a rebound effect through metabolic imbalances, including inflammation or hormonal disruption. Animal data show exercise at 60–70% VO₂max activates AMPK, suppresses ACC, and upregulates LDL receptors, accelerating LDL-C clearance [39–41]. Exercise-induced DNA demethylation of the lipoprotein lipase (LPL) promoter may further enhance lipid metabolism via lasting epigenetic changes [42, 43].

Sociodemographic patterns indicate barriers to higher PA: participants with higher education were overrepresented in low-activity groups, possibly due to cognitively demanding work reducing exercise opportunities [44]. Such roles may over activate the default mode network (DMN), lowering striatal dopamine D2 receptor expression and inducing “decision fatigue,” reducing exercise motivation [45–47]. Higher-activity groups more often received physician exercise advice, potentially creating a “positive feedback loop,” while low-activity individuals risk a “guidance deficit–risk accumulation” cycle [48].Functional advantages in high-activity groups may reflect neuromuscular adaptations such as cerebellar–basal ganglia remodeling, improving motor coordination and gait stability [49, 50].

Subgroup analyses indicated that moderate-to-vigorous physical activity (MVPA) was consistently associated with lower dyslipidemia risk across demographic groups. Nationally, the prevalence of dyslipidemia in men and women during the study period was notably lower than rates reported by Mutalifu et al. in Xinjiang [51], a difference likely attributable to regional dietary habits, the predominance of rural populations, and occupational activity patterns. Although rural residents constituted the majority of the MVPA group, their risk reduction was comparable to that observed in urban participants. Prior research in China attributes this parity to the sustained nature of agricultural labor, which promotes greater lipid oxidation efficiency than the more intermittent activity typical of urban environments, albeit with a potential for chronic inflammation from repetitive motions [52, 53].

The protective association of MVPA with dyslipidemia was evident across age, sex, and residential categories, with no significant interaction effects, suggesting broad applicability. However, attenuation of this effect among individuals with elevated BMI points to possible metabolic resistance in overweight or obese populations—potentially mediated by insulin resistance, chronic low-grade inflammation, or adaptive changes in energy balance that blunt exercise-induced lipid benefits. Such heterogeneity highlights the importance of tailoring exercise prescriptions to individual metabolic phenotypes [54–56].

When lipid subcomponents were examined separately, distinct dose–response patterns emerged. High-density lipoprotein cholesterol (HDL-C) showed the strongest positive association with physical activity, increasing progressively from the least to the most active participants, with significant differences once activity exceeded minimal levels. The steady decline in HDL-C deficiency aligns with previous evidence linking physical activity to ApoA1 upregulation and improved HDL functionality [57–59]. Low-density lipoprotein cholesterol (LDL-C) declined in a graded fashion, with the largest improvement occurring between sedentary individuals and those engaging in moderate levels of activity, consistent with findings that moderate-intensity exercise can substantially reduce atherosclerosis risk [60, 61]. Beyond this point, further gains were limited, suggesting a threshold rather than a strictly linear effect.

For total cholesterol (TC), notable reductions appeared only when moving from sedentary to low–moderate activity levels, consistent with the proposal that TC may act as an early-phase sensitive biomarker [62]. Although overall prevalence of TC abnormalities decreased, a slight rebound at higher activity volumes hints at non-linear modulation. Triglycerides (TG) exhibited a less consistent relationship, with significant improvement only at the highest activity volumes. This pattern suggests that more vigorous or sustained exercise may be necessary for optimal TG control, echoing earlier reports on the limitations of moderate activity [28, 63]. The early decline in TG abnormalities further implies an acute responsiveness to the transition from sedentary to active lifestyles, likely mediated by exercise-induced activation of lipoprotein lipase [30].

These lipid-specific patterns challenge the notion that “more is always better” and instead point toward differentiated PA targets: sustained moderate activity for LDL-C control, higher volumes for TC reduction, and flexible but progressive dosing for optimizing HDL-C and TG. Incorporating both subgroup variability and lipid-specific responsiveness into clinical and public health recommendations could enhance the precision and efficiency of exercise-based strategies for dyslipidemia management.

### Limitations

This study has several limitations. Although the CHARLS cohort is nationally representative, exclusions were necessary to ensure data completeness, and residual selection bias remains possible despite multiple imputation for missing data. Physical activity was self-reported using IPAQ-derived questionnaires, which are practical for large-scale studies but prone to recall bias. Device-based monitoring was not implemented to maintain long-term follow-up, but future work could combine accelerometer and self-report methods.

Although extensive covariate adjustments were made, unmeasured confounding—particularly dietary intake and genetic predisposition—may have influenced results. For example, active individuals may also maintain healthier diets, while high-fat intake could offset exercise benefits.

Finally, in calculating MET-h/week, daily activity duration was truncated at four hours per intensity level to reduce bias from extreme values and ensure comparability, but this may underestimate activity in highly active individuals such as manual laborers.

## Conclusion

This 10-year cohort study of adults aged 45 and older from the CHARLS dataset reveals a nonlinear (S-shaped) relationship between physical activity (PA) and dyslipidemia risk, with optimal protection occurring at 63.88–163.66 MET-h/week. From a public health standpoint, we recommend that middle-aged and older adults engage in approximately 8–20 h of moderate-intensity activity (e.g., brisk walking, household tasks, active commuting) or 4–10 h of vigorous-intensity activity (e.g., running, heavy labor, sports) per week, incorporating both occupational and leisure activities, to achieve optimal lipid control and reduce long-term dyslipidemia risk. Beyond this activity range, benefits plateau, and excessive physical activity may result in physical strain.

The effects of physical activity were lipid-specific: HDL-C improved continuously with increased PA, LDL-C reductions plateaued at moderate PA levels, while triglycerides (TG) and total cholesterol (TC) showed modest, nonlinear changes, suggesting differing sensitivities to PA dose. Subgroup analyses revealed consistent protective effects across age, sex, and region; however, these effects were attenuated in individuals with higher BMI or unmarried status, underscoring the role of metabolic and social factors.

These findings emphasize the need for tailored exercise guidelines that consider both lipid-specific responses and demographic factors. Precision exercise recommendations could offer a more effective and personalized approach to cardiovascular risk reduction in aging populations.

## Data Availability

The data analyzed in this study are from the China Health and Retirement Longitudinal Study (CHARLS), which is publicly available at http://charls.pku.edu.cn. Researchers can apply for access to the dataset through the official website. The authors do not have any special access privileges to the data and others can obtain it in the same manner.
